# Supplementary material for: Genetic diversity in the IZUMO1-JUNO protein-receptor pair involved in human reproduction
Source: PLoS One. 2021 Dec 8;16(12):e0260692. doi: 10.1371/journal.pone.0260692 (PMC8654184; doi:10.1371/journal.pone.0260692)

Figure S1: Histograms of the frequencies of FST values between all 26 regional populations for the IZUMO1 gene between A) all 2504 individuals sampled in the 1000 Genomes Project, B) just the males sampled and C) just the females sampled. The red line indicates the 0.102 reference value for average human genome-wide FST(22). The maximum FST value was 0.503 between the YRI and CDX populations. For just the male population the maximum FST value was 0.654 between the YRI and CDX populations. For just the female population the maximum FST value was 0.528 between the MSL and CDX populations. These values were calculated using all SNPs with a MAF of 1% or greater.

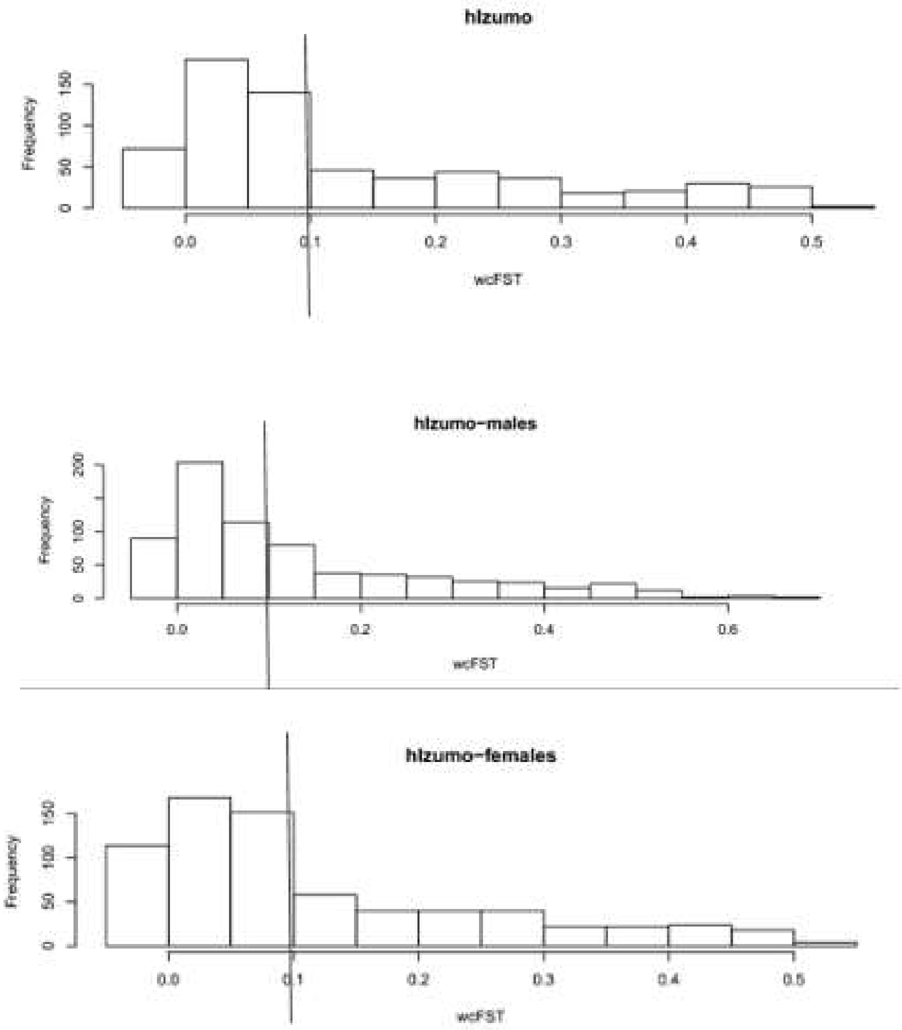

Supplement: S1 Fig — (PDF) [file pone.0260692.s001.pdf]
