# Supplementary material for: Genetic diversity in the IZUMO1-JUNO protein-receptor pair involved in human reproduction
Source: PLoS One. 2021 Dec 8;16(12):e0260692. doi: 10.1371/journal.pone.0260692 (PMC8654184; doi:10.1371/journal.pone.0260692)

Figure S2: Histograms of the frequencies of FST values between all 26 regional populations for the JUNO gene between A) all 2504 individuals sampled in the 1000 Genomes Project, B) just the males sampled and C) just the females sampled. The red line indicates the 0.102 reference value for average human genome-wide FST(22). The maximum FST value was 0.372 between the MSL and CDX populations. For just the male population the maximum FST value was 0.393 also between the MSL and CDX populations. For just the female population the maximum FST value was 0.528 between the MSL and CDX populations. These values were calculated using all of the SNPs with a MAF of 1% or greater.

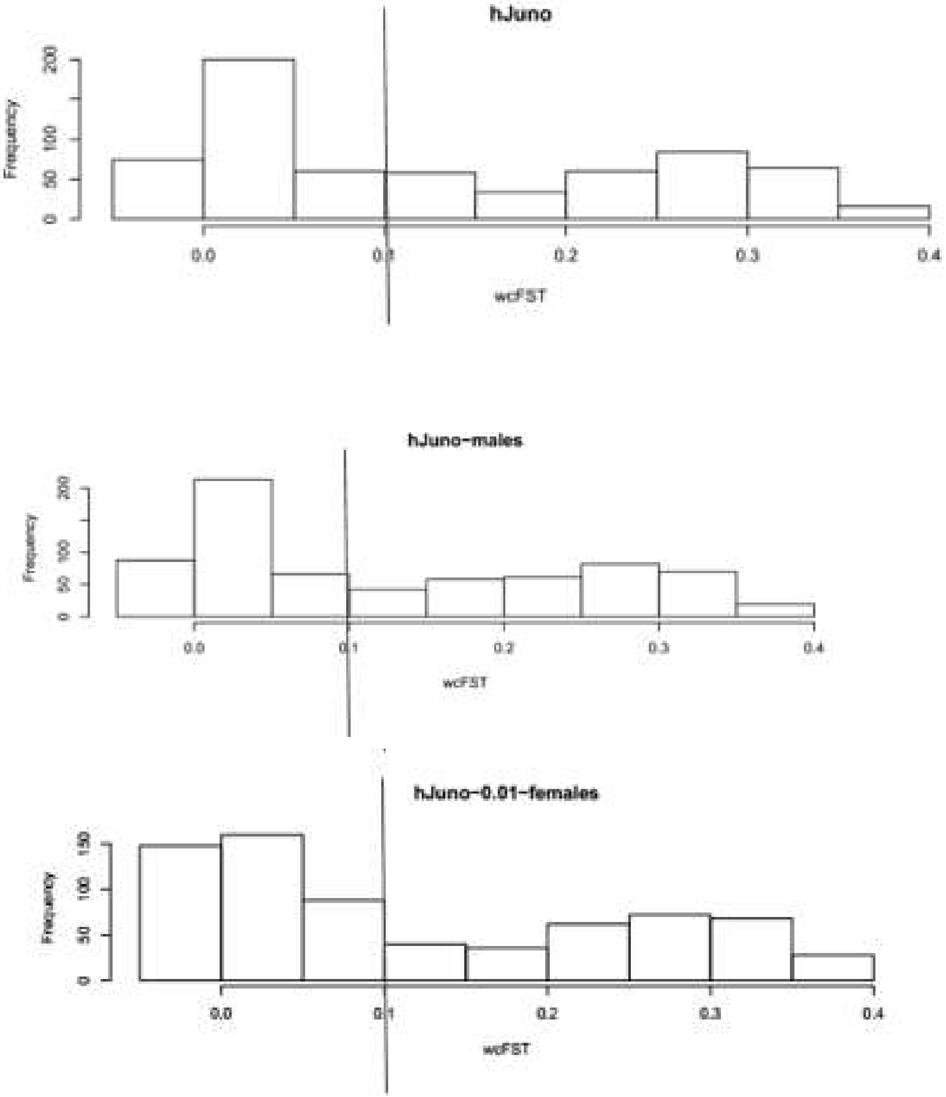

Supplement: S2 Fig — (PDF) [file pone.0260692.s002.pdf]
