# Supplementary material for: Genetic diversity in the IZUMO1-JUNO protein-receptor pair involved in human reproduction
Source: PLoS One. 2021 Dec 8;16(12):e0260692. doi: 10.1371/journal.pone.0260692 (PMC8654184; doi:10.1371/journal.pone.0260692)

Figure S3: Principal Component analysis of FST values between population groups for human IZUMO1 calculated for the entire set of 2,504 individuals sampled in the 1000 Genomes project. Squares are populations categorized in the supergroup AFR (African); crosses are EUR (European); circles are EAS (East Asian); stars are SAS (South Asian); triangles are AMR (American). The population designations follow the 1000 Genome project annotations, as indicated in Table S3. For human Izumo1, the supergroups AFR and EAS segregate from the others. Components 1 and 2 carry 99% of the information contained in the pairwise FST values and are, thus, effective proxies of the genetic diversity between the populations studied.

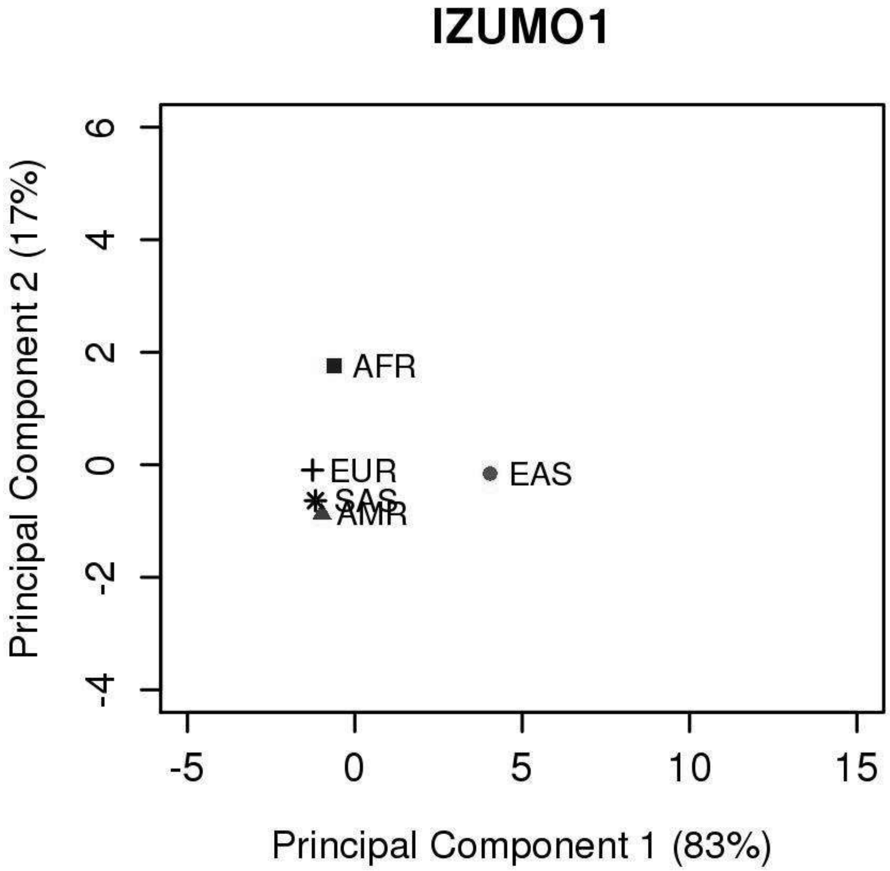

Supplement: S3 Fig — (PDF) [file pone.0260692.s003.pdf]
