# Supplementary material for: Genetic diversity in the IZUMO1-JUNO protein-receptor pair involved in human reproduction
Source: PLoS One. 2021 Dec 8;16(12):e0260692. doi: 10.1371/journal.pone.0260692 (PMC8654184; doi:10.1371/journal.pone.0260692)

Figure S5: Scanning for signals of positive selection on chromosome 11 of ALL individuals sequenced in the 1000 Genomes project. Regions with μ scores above the 99.95% threshold (solid red line) are expected to be under positive selection. This threshold is based on all μ scores for a dataset and it is, hence, population-dependent. The genomic region corresponding to the JUNO gene is marked by green dashed lines. the genomic region corresponding to JUNO is within a region of μ values modestly above the threshold set and borders a region of high μ values. This suggests that the indicators of positive selection we identified in JUNO from the analysis of nucleotide diversity, Tajima's D, and haplotype inference could have originated from a hitchhiking effect of a selective sweep (23).

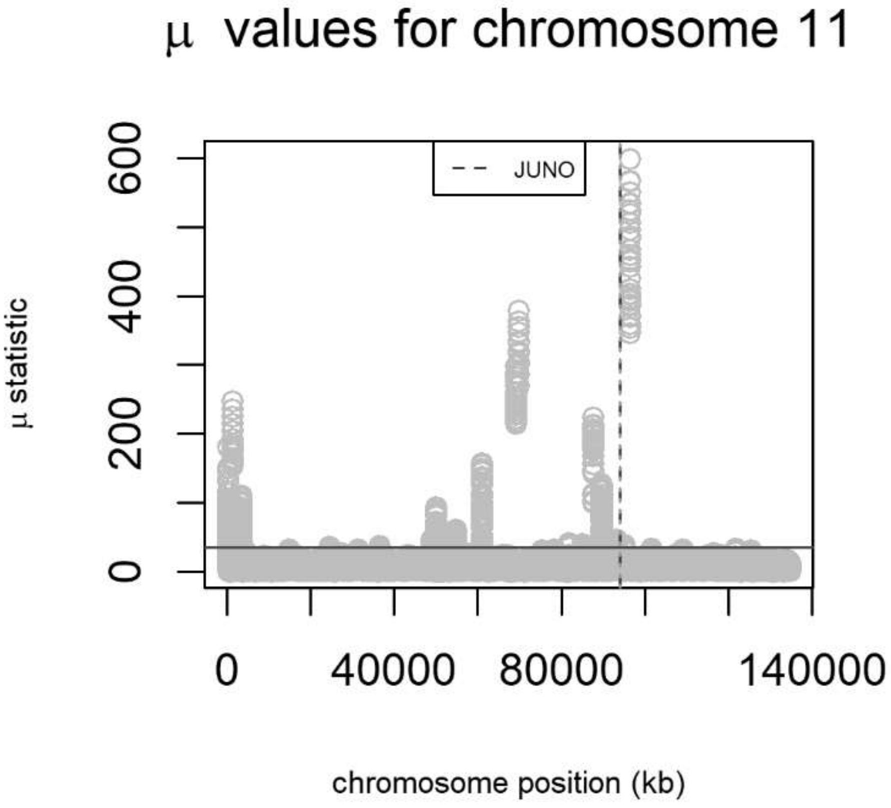

Supplement: S5 Fig — (PDF) [file pone.0260692.s005.pdf]
