# Supplementary material for: Genetic diversity in the IZUMO1-JUNO protein-receptor pair involved in human reproduction
Source: PLoS One. 2021 Dec 8;16(12):e0260692. doi: 10.1371/journal.pone.0260692 (PMC8654184; doi:10.1371/journal.pone.0260692)
Supplement: S1 Table — (PDF) [file pone.0260692.s006.pdf]

Table S1: Comprehensive breakdown of the variants in the IZUMO1 gene sequence when unfiltered and filtered with a minor allele frequency (MAF) of 5% using SNPEff (1).

	No maf filtering	Maf 5% frequency
Variants	192	31
Variant rates	307963	1907386
SNPs	189	31
Insertions	1	0
Deletions	2	0
Low Impact Effects	199 (29.4%)	23 (21.5%)
Moderate Impact Effects	11 (1.6%)	1 (0.9%)
Modifier Impact Effects	466 (68.9%)	83 (77.6%)
Missense Mutations	12 (57.1%)	1 (33.3%)
Silent Mutations	9 (42.9%)	2 (66.7%)
Downstream Effects	143 (21.2%)	29 (27.1%)
Intergenic Effects	20 (3.0%)	6 (5.6%)
Intragenic Effects	1 (0.1%)	0
Intron Effects	132 (19.5%)	18 (16.8%)
Next Protein Effects	182 (26.9%)	21 (19.6%)
Non-synonymous Coding Effects	11 (1.6%)	1 (0.9%)
Non-synonymous Start Effects	1 (0.1%)	0
Splice Site Region and Intron Effect	5 (0.7%)	0
Start Gained Effect	2 (0.3%)	0
Synonymous Coding Effect	9 (1.3%)	2 (1.9%)
Upstream Effects	157 (23.3%)	26 (24.3%)
UTR 5 Prime Effect	13 (1.9%)	4 (3.7%)
