# Supplementary material for: Genetic diversity in the IZUMO1-JUNO protein-receptor pair involved in human reproduction
Source: PLoS One. 2021 Dec 8;16(12):e0260692. doi: 10.1371/journal.pone.0260692 (PMC8654184; doi:10.1371/journal.pone.0260692)
Supplement: S2 Table — (PDF) [file pone.0260692.s007.pdf]

Table S2: Comprehensive breakdown of the variants in the JUNO gene sequence when unfiltered and filtered with a MAF of 5% using SNPEff (1).

	No maf filtering	Maf 5% frequency
Variants	90	7
Variant rates	1500072	19286645
SNPs	82	7
Mixed Variants	8	0
High Impact Effects	3 (1.8%)	0
Low Impact Effects	86 (51.1%)	3 (30%)
Moderate Impact Effects	19 (11.3%)	1 (10%)
Modifier Impact Effects	60 (35.7%)	6 (60%)
Missense Mutations	18 (60%)	1 (100%)
Silent Mutations	3 (10%)	0
Downstream Effects	9 (30%)	0
Intergenic Effects	4 (2.4%)	0
Intron Effects	53 (31.5%)	5 (50%)
Next Protein Effects	78 (46.4%)	3 (30%)
Non-synonymous Coding Effects	18 (10.7%)	1 (10%)
Stop Gained Effects	3 (1.8%)	0
Synonymous Coding Effect	9 (5.4%)	0
UTR 5 Prime Effect	3 (1.8%)	1 (10%)
