# Supplementary material for: Genetic diversity in the IZUMO1-JUNO protein-receptor pair involved in human reproduction
Source: PLoS One. 2021 Dec 8;16(12):e0260692. doi: 10.1371/journal.pone.0260692 (PMC8654184; doi:10.1371/journal.pone.0260692)
Supplement: S3 Table — (PDF) [file pone.0260692.s008.pdf]

Table S3: A list of the 26 different populations sampled by the 1000 Genomes project(2) clustered into five larger population groups, where *n* signifies the number of individuals in each population group.

**Category**	**n**	**Populations included:**	**Population Code**
South Asia	489	Bengali in Bangladesh<br>Gujarati Indian<br>Indian Telugu in the UK<br>Punjabi in Lahore,Pakistan pjl<br>Sri Lankan Tamil in the UK	BEB<br>GIH<br>ITU<br>PJL<br>STU
East Asian	504	Japanese in Tokyo, Japan<br>Han Chinese in Bejing,<br>China Southern Han Chinese, China<br>Chinese Dai in Xishuangbanna<br>Kinh in Ho Chi Minh City, Vietnam	JPT<br>CHB<br>CHS<br>CDX<br>KHV
Europe	503	Northern and Western European Finnish in Finland<br>Finnish in Finland<br>British in England and Scotland<br>Iberian populations in Spain<br>Toscani in Italia	CEU<br>FIN<br>GBR<br>IBS<br>TSI
America	347	Colombian in Medellin, Colombia<br>Mexican Ancestry in Los Angeles,<br>Peruvian in Lima, Peru<br>Puerto Rican in Puerto Rico	CLM<br>MXL<br>PEL<br>PUR
Africa	661	African Caribbean in Barbados<br>African Ancestry in Southwest US<br>Esan in Nigeria<br>Gambian in Western Division<br>Luhya in Webuye, Kenya<br>Mende in Sierra Leone<br>Yoruba in Ibadan, Nigeria	ACB<br>ASW<br>ESN<br>GWD<br>LWK<br>MSL<br>YRI
