# Supplementary material for: Genetic diversity in the IZUMO1-JUNO protein-receptor pair involved in human reproduction
Source: PLoS One. 2021 Dec 8;16(12):e0260692. doi: 10.1371/journal.pone.0260692 (PMC8654184; doi:10.1371/journal.pone.0260692)
Supplement: S4 Table — African versus Non-African populations. (PDF) [file pone.0260692.s009.pdf]

Table S4: Tajima's D analysis of various genes under different types of selection(3-8). Population sizes (n) are reported in parenthesis. Tajima's D was calculated using VCFTools(9) in bins of 100 bp for all biallelic sites within the location range of each gene.

**Selection**	**Gene**	**Location (GRCh37.p13)**	**Tajima's D all populations (n=2,504)**	**Literature Value**	**Reference**
Unknown					
	IZUMO1	Chr 9 49244073-49250831	-0.35532	N/A	N/A
	JUNO	Chr 11 94038803-94040858	-0.77916	N/A	N/A
Neutral					
	LTA	Chr 6 31539876-31542101	-0.45138	0.746 (n=282) 6 Chinese populations	(5)
	TAS2R38	Chr 7 141463897-141464997	-0.58725	1.078 (n=8,589)	(10)
	TBX1	Chr 22 19744226-19771116	-0.69686	-0.25 (n=124) (22 = EUR, 27 = AFR, 24 = ASI, 22 = AMR)	(11)
	VTN	Chr 17 26694298-26697373	-0.61777	Value not reported	
Balancing					
	ABO	Chr 9 136130563 -136150630	-0.07299	2.035 EUR (n=23) 1.772 AFR (n=24)	(4, 6) (12)
	BPIFB4	Chr 20 31669318-316699557	-0.56074	Value not reported	(7)

	BTN1A1	Chr 6 26500577-26510653	-0.57767	Value not reported	(7)
	CDSN	Chr 6 31082865-31088252, complement	0.333637	Value not reported	(7)
	CLCNKB	Chr 10 16370231-16383821	-0.34419	Value not reported	(7)
	ERAP2	Chr 5 96211644-96255420	-0.30469	1.526 (n=180)	(3)
	GRIN3A	Chr 9 104331634-104500862, complement	-0.53335	Value not reported	(7)
	HLAA	Chr 6 29910247-29913661	0.656452	2.9 (n=205)	(7, 13, 14)
	HLAB	Chr 6 31321649-31324989, complement	0.354656	2.4 (n=205)	(7, 13, 14)
	KRT6C	Chr 12 52862300-52867569, complement	-0.32544	Value not reported	(7)
	KRT84	Chr 12 52771596-52779417, complement	-0.34531	Value not reported	(7)
	TRIM22	Chr 11 5710817 - 5732093	-0.37645	Value not reported	(7)
Positive					
	ABHD1	Chr 2 27346632-27353680	-0.72632	Value not reported	(8)
	ALMS1	Chr 2 73612886-73837047	-0.67787	Value not reported	(8)
	APOBEC3F	Chr 22 39436609-39451977	-0.67458	Value not reported	(8)
	APOBEC3G	Chr 22 39473010-39483748	-0.67032	Value not reported	(8)

	CD36	Chr 7 80231504-80308593	-0.5766	Value not reported	(8)
	CD58	Chr 1 117057156-117113715, complement	-0.70801	Value not reported	(8)
	CD72	Chr 9 35609976-35618862, complement	-0.7711	Value not reported	(8)
	EDAR	Chr 2 109510927-109605828	-0.66222	Value not reported	(8, 15)
	FAF1	Chr 1 50906935-51425936, complement	-0.73988	Value not reported	(8)
	GRAP2	Chr 22 40297086-40369347	-0.7289	Value not reported	(8)
	HYAL3	Chr 3 50330259-50336899, complement	-0.73139	Value not reported	(8)
	ITGAE	Chr 17 3617919-3704537, complement	-0.57616	Value not reported	(8)
	KEL	Chr 7 142638201-142659503	-0.76554	-2.467 EUR (n=23)<br>-0.823 AFR (n=24)	(6)
	LCT	Chr 2 136545415-136594750, complement	-0.61215	Value not reported	(16)
	NPAP1	Chr 15 24920541-24928593	-0.74745	Value not reported	(8)
	PRM1	Chr 16 11374693-11375192, complement	-0.84476	Value not reported	(8)
	PRM2	Chr 16 11369493-113	-0.91328	Value not reported	(8)

		70337, complement			
	PROS1	Ch3 3 93591881-936 92934, complement	-0.80824	-1.44 (n = 47) (24 = AFR, 23 = EUR)	(17)
	RBAK	Chr 7 5085452-5112 854	-0.62683	Value not reported	(8)
	RHCE	Chr 1 25687853-257 47363, complement	-0.63686	Value not reported	(8)
	SLC24A5	Chr 15 48413169-484 34926	-0.73307	Value not reported	(8)
	SYT1	Chr 12 79257773-798 45788	-0.66917	Value not reported	(8)
	TRPV6	Chr 7 142568956-142583490	-0.67565	-2.865 EUR (n=23) 0.893 AFR (n=24)	(6, 18, 19)
