# Supplementary material for: Genetic diversity in the IZUMO1-JUNO protein-receptor pair involved in human reproduction
Source: PLoS One. 2021 Dec 8;16(12):e0260692. doi: 10.1371/journal.pone.0260692 (PMC8654184; doi:10.1371/journal.pone.0260692)
Supplement: S5 Table — (PDF) [file pone.0260692.s010.pdf]

Table S5: Hardy-Weinberg Equilibrium analysis of IZUMO1 gene in males only and the entire population in each of the groups included in the analyzed haplotype(20).

Location	rs2307018		rs2307019		rs838148	
Population	All	Males	All	Males	All	Males
AFR	0.7809	1	0.7809	1	0.8287	0.9697
AMR	0.9762	0.57	0.9762	0.57	**0.9762**	**0.2741**
EUR	0.3691	1	0.3691	1	0.8109	0.428
EAS	0.8668	0.7768	0.8668	0.7768	0.9064	1.00
SAS	0.3308	0.2600	0.3308	0.2600	1	0.8749
ASI	**5.83E-06**	**6.91E-05**	**5.83E-06**	**6.91E-05**	0.3669	0.3098
ALL	2.06E-13	4.62E-08	2.06E-13	4.62E-08	0.0039	0.0025
