# Supplementary material for: Genetic diversity in the IZUMO1-JUNO protein-receptor pair involved in human reproduction
Source: PLoS One. 2021 Dec 8;16(12):e0260692. doi: 10.1371/journal.pone.0260692 (PMC8654184; doi:10.1371/journal.pone.0260692)
Supplement: S6 Table — (PDF) [file pone.0260692.s011.pdf]

Table S6: Hardy-Weinberg Equilibrium analysis of JUNO gene in females only and the entire population in each of the groups included in the analyzed haplotype(20).

Location	rs61742524		rs55784852		rs16920146		rs7925833		rs7935583	
Population	All	Females	All	Females	All	Females	All	Females	All	Females
AFR	**0.4875**	**0.0221**	**0.4875**	**0.022**	**0.4875**	**0.0221**	**0.666**	**0.1764**	**0.4875**	**0.0221**
AMR	**0.491**	**0.1201**	**0.491**	**0.120**	**0.491**	**0.1201**	**0.4049**	**0.0725**	**0.491**	**0.1201**
EUR	1	1	1	1	1	1	1	1	1	1
EAS	1	1	1	1	1	1	1	1	1	1
SAS	1	1	1	1	1	1	1	1	1	1
ASI	1	1	1	1	1	1	1	1	1	1
ALL	1.36E-20	4.25E-10	5.26E-20	4.25E-10	5.26E-20	4.25E-10	1.17E-20	009.28E-06	1.78E-20	4.25E-10
