# Supplementary material for: Genetic diversity in the IZUMO1-JUNO protein-receptor pair involved in human reproduction
Source: PLoS One. 2021 Dec 8;16(12):e0260692. doi: 10.1371/journal.pone.0260692 (PMC8654184; doi:10.1371/journal.pone.0260692)
Supplement: S7 Table — (PDF) [file pone.0260692.s012.pdf]

Table S7: FST values in the IZUMO1 gene between the five larger population groups for the entire set of 2504 individuals sampled in the 1000 Genomes project. These FST values were calculated using SNPs with a MAF of at least 1%. For comparison, a genome wide FST value for the human genome is 0.12. The average of all pairwise values is 0.150.

	EUR	EAS	AMR	SAS	AFR
EUR		0.296	0.023	0.020	0.080
EAS	0.296		0.196	0.224	0.447
AMR	0.023	0.196		0.004	0.123
SAS	0.020	0.224	0.004		0.085
AFR	0.080	0.447	0.123	0.085	
