# Supplementary material for: Genetic diversity in the IZUMO1-JUNO protein-receptor pair involved in human reproduction
Source: PLoS One. 2021 Dec 8;16(12):e0260692. doi: 10.1371/journal.pone.0260692 (PMC8654184; doi:10.1371/journal.pone.0260692)
Supplement: S8 Table — (PDF) [file pone.0260692.s013.pdf]

Table S8: FST values in the JUNO gene between the five larger population groups for the entire set of 2504 individuals sampled in the 1000 Genomes project. These FST values were calculated using SNPs with a MAF of at least 1%. For comparison, a genome wide FST value for the human genome is 0.12. The average of all pairwise values is 0.135.

	EUR	EAS	AMR	SAS	AFR
EUR		0.001	0.007	0.114	0.310
EAS	0.001		0.004	0.096	0.304
AMR	0.007	0.004		0.063	0.247
SAS	0.114	0.096	0.063		0.203
AFR	0.310	0.304	0.247	0.203	
