# Supplementary material for: Genetic diversity in the IZUMO1-JUNO protein-receptor pair involved in human reproduction
Source: PLoS One. 2021 Dec 8;16(12):e0260692. doi: 10.1371/journal.pone.0260692 (PMC8654184; doi:10.1371/journal.pone.0260692)
Supplement: S9 Table — (PDF) [file pone.0260692.s014.pdf]

Table S9: Percent identities in reference to the IZUMO1 *Homo sapiens* amino acid sequence for 29 homologous mammalian species. The E-value indicates the statistical significance of the data, the smaller the number the better, and the query cover indicates the percentage of the sequence that overlaps with the *Homo sapiens* sequence. The % identity BLAST is generated by a local alignment and were acquired from http://www.ncbi.nlm.nih.gov.The % identity ClustalX is generated by global alignment and was calculated using ClustalX(21).

**Species**	**Common Name**	**E-value**	**Query Cover**	**% Identity BLAST**	**% Identity Clustalx**
Homo sapiens	Human	2.00E-164	100	100	100
Pan troglodytes	Chimpanzee	4.00E-92	100	100	99
Gorilla gorilla gorilla	Lowland Gorilla	3.00E-161	100	99	98
Nomascus leucogenys	White-cheeked gibbon	3.00E-155	100	95	94
Papio Anubis	Baboon	6.00E-148	100	92	91
Mandrillus leucophaeus	Drill	2.00E-147	100	92	91
Macaca fascicularis	Long-tailed macaque	3.00E-148	100	92	89
Macaca nemestrina	Pigtail monkey	4.00E-150	100	92	92
Rhinopithecus roxellana	Golden snub-nosed monkey	3.00E-146	100	91	91
Colobus angolensis palliates	Peter's Angola Colobus	3.00E-147	100	92	91
Saimiri boliviensis boliviensis	Black-headed squirrel monkey	3.00E-125	94	83	81
Aotus nancymaae	Nancy Ma's night monkey	4.00E-129	100	82	84
Propithecus coquereli	Coqueral's sifaka	7.00E-108	98	71	70
Felis catus	Cat	9.00E-76	96	58	50
Microcebus murinus	Gray mouse lemur	8.00E-87	98	59	64
Equus przewalskii	Przewalski horse	1.00E-89	97	66	59
Pteropus vampyrus	Large flying fox (bat)	8.00E-80	100	56	58
Otolemur garnettii	Northern Greater Galago	4.00E-81	82	56	56
Loxodonta Africana	African savannah elephant	2.00E-84	74	72	56
Camelus dromedaries	One-humped camel	2.00E-94	100	63	65
Trichechus manatus latirostris	West indian manatee	2.00E-84	67	76	55
Orcinus orca	Orca	1.00E-96	99	64	65
Heterocephalus glaber	Naked mole rat	5.00E-85	100	60	55
Microtus ochrogaster	Prairie voles	5.00E-80	75	63	52
Chinchilla lanigera	Long-tailed chinchilla	1.00E-85	100	64	59
Jaculus jaculus	Lesser Egyptian Jerboa	1.00E-79	71	67	53
Rattus norvegicus	Rat	1.00E-79	75	63	55
Octodon degus	Degu	1.00E-82	73	69	55
Bos mutus	Wild yak	1.00E-84	89	64	60
Mus musculus	Mouse	1.00 E-81	78	46	47
