# Supplementary material for: Genetic diversity in the IZUMO1-JUNO protein-receptor pair involved in human reproduction
Source: PLoS One. 2021 Dec 8;16(12):e0260692. doi: 10.1371/journal.pone.0260692 (PMC8654184; doi:10.1371/journal.pone.0260692)
Supplement: S10 Table — (PDF) [file pone.0260692.s015.pdf]

Table S10: Percent identities in reference to the JUNO *Homo sapiens* amino acid sequence for 29 homologous mammalian species. The E-value indicates the statistical significance of the data, the smaller the number the better, and the query cover indicates the percentage of the sequence that overlaps with the *Homo sapiens* sequence. The % identity BLAST is generated by a local alignment and were acquired from http://www.ncbi.nlm.nih.gov.The % identity ClustalX is generated by global alignment and was calculated using ClustalX(21).

**Species**	**Common Name**	**E-value**	**Query Cover**	**% Identity BLAST**	**% Identity Clustalx**
Homo sapiens	Human	7.00E-173	100	100	100
Pan troglodytes	Chimpanzee	1.00E-163	95	100	99
Gorilla gorilla gorilla	Lowland Gorilla	2.00E-163	95	99	99
Nomascus leucogenys	White-cheeked gibbon	1.00E-160	95	98	97
Papio Anubis	Baboon	2.00E-159	95	97	96
Mandrillus leucophaeus	Drill	5.00E-159	95	97	96
Macaca fascicularis	Long-tailed macaque	5.00E-158	95	96	96
Macaca nemestrina	Pigtail monkey	3.00E-158	95	96	95
Rhinopithecus roxellana	Golden snub-nosed monkey	6.00E-156	93	97	95
Colobus angolensis palliates	Peter's Angola Colobus	1.00E-152	93	96	94
Saimiri boliviensis boliviensis	Black-headed squirrel monkey	6.00E-150	95	92	92
Aotus nancymaae	Nancy Ma's night monkey	9.00E-148	95	90	90
Propithecus coquereli	Coqueral's sifaka	8.00E-145	100	87	86
Felis catus	Cat	4.00E-132	99	77	77
Microcebus murinus	Gray mouse lemur	2.00E-131	100	80	82
Equus przewalskii	Przewalski horse	5.00E-122	95	77	77
Pteropus vampyrus	Large flying fox (bat)	3.00E-121	94	78	79
Otolemur garnettii	Northern Greater Galago	2.00E-119	94	76	75
Loxodonta Africana	African savannah elephant	1.00E-116	94	76	76
Camelus dromedaries	One-humped camel	7.00E-116	93	78	76
Trichechus manatus latirostris	West indian manatee	4.00E-116	92	76	77
Orcinus orca	Orca	5.00E-115	94	76	74
Heterocephalus glaber	Naked mole rat	5.00E-115	99	71	73
Microtus ochrogaster	Prairie voles	4.00E-112	98	73	74
Chinchilla lanigera	Long-tailed chinchilla	2.00E-112	100	71	72
Jaculus jaculus	Lesser Egyptian Jerboa	8.00E-109	99	72	71
Rattus norvegicus	Rat	1.00E-105	98	69	69
Octodon degus	Degu	1.00E-101	98	66	67
Bos mutus	Wild yak	6.00E-101	88	72	68
Mus musculus	Mouse	8.00E-103	99	67	66
