# Supplementary material for: Genetic diversity in the IZUMO1-JUNO protein-receptor pair involved in human reproduction
Source: PLoS One. 2021 Dec 8;16(12):e0260692. doi: 10.1371/journal.pone.0260692 (PMC8654184; doi:10.1371/journal.pone.0260692)
Supplement: S11 Table — (PDF) [file pone.0260692.s016.pdf]

Table S11: The average, maximum and minimum percent identity values for both JUNO and IZUMO1 nucleotide sequences for all of the 2504 individuals in reference to the GRCh37 reference genome used in the 1000 Genomes project.

**Protein**	**JUNO**	**IZUMO1**
Average %ID	99.89	99.50
Max %ID	100.00	100.00
Min %ID	99.49	99.25
Standard Deviation	0.11	0.24
