# Supplementary material for: Genetic diversity in the IZUMO1-JUNO protein-receptor pair involved in human reproduction
Source: PLoS One. 2021 Dec 8;16(12):e0260692. doi: 10.1371/journal.pone.0260692 (PMC8654184; doi:10.1371/journal.pone.0260692)
Supplement: S12 Table — (PDF) [file pone.0260692.s017.pdf]

Table S12: A description of the synonymous and non-synonymous SNPs in the IZUMO1 gene when filtered by a MAF of 5%.

SNP	rs2307018	rs2307019	rs8108468
Effect	Synonymous Coding	Non-synonymous Coding	Synonymous Coding
	Next Protein Effect	Next Protein Effect	Next Protein Effect
Impact	Low Impact	Moderate Impact	Low Impact
Location	Topological Domain: Cytoplasmic	Topological Domain: Cytoplasmic	Topological Domain: Extracellular
Amino Acid	A333	A33V	F107
	Upstream Modifier (RASIP1)	Upstream Modifier (RASIP1)	Upstream Modifier (RASIP1)
			Downstream Modifier (FUT1)
Type of Mutation	Silent Mutation	Missense Mutation	Silent Mutation
