# Supplementary material for: Genetic diversity in the IZUMO1-JUNO protein-receptor pair involved in human reproduction
Source: PLoS One. 2021 Dec 8;16(12):e0260692. doi: 10.1371/journal.pone.0260692 (PMC8654184; doi:10.1371/journal.pone.0260692)
Supplement: S13 Table — (PDF) [file pone.0260692.s018.pdf]

Table S13: A description of the synonymous and non-synonymous SNPs in the JUNO gene when filtered by a MAF of 5%.

SNP	rs61742524
Effect	Non-synonymous Coding<br>Next Protein Effect
Impact	Moderate Impact
Location	Topological Domain: Cytoplasmic
Amino Acid	C3W
Type of Mutation	Missense Mutation
