# Supplementary material for: Genetic diversity in the IZUMO1-JUNO protein-receptor pair involved in human reproduction
Source: PLoS One. 2021 Dec 8;16(12):e0260692. doi: 10.1371/journal.pone.0260692 (PMC8654184; doi:10.1371/journal.pone.0260692)
Supplement: S1 References — (PDF) [file pone.0260692.s019.pdf]

References

1. Cingolani P, Platts A, Wang le L, Coon M, Nguyen T, Wang L, et al. A program for annotating and predicting the effects of single nucleotide polymorphisms, SnpEff: SNPs in the genome of Drosophila melanogaster strain w1118; iso-2; iso-3. Fly (Austin). 2012;6(2):80-92.
2. 1000 Genomes Project Consortium AG, Auton A, Brooks LD, DePristo MA, Durbin RM et al. An integrated map of genetic variation from 1,092 human genomes. Nature. 2012;491(7422):56-65.
3. Andres AM, Dennis MY, Kretzschmar WW, Cannons JL, Lee-Lin SQ, Hurle B, et al. Balancing selection maintains a form of ERAP2 that undergoes nonsense-mediated decay and affects antigen presentation. PLoS Genet. 2010;6(10):e1001157.
4. Carlson CS, Thomas DJ, Eberle MA, Swanson JE, Livingston RJ, Rieder MJ, et al. Genomic regions exhibiting positive selection identified from dense genotype data. Genome Res. 2005;15(11):1553-65.
5. Zhang Y, Zhang F, Lin H, Shi L, Wang P, Shi L, et al. Nucleotide polymorphism of the TNF gene cluster in six Chinese populations. J Hum Genet. 2010;55(6):350-7.
6. Akey JM, Eberle MA, Rieder MJ, Carlson CS, Shriver MD, Nickerson DA, et al. Population history and natural selection shape patterns of genetic variation in 132 genes. PLoS Biol. 2004;2(10):e286.
7. Andres AM, Hubisz MJ, Indap A, Torgerson DG, Degenhardt JD, Boyko AR, et al. Targets of balancing selection in the human genome. Mol Biol Evol. 2009;26(12):2755-64.
8. Sabeti PC, Schaffner SF, Fry B, Lohmueller J, Varilly P, Shamovsky O, et al. Positive natural selection in the human lineage. Science. 2006;312(5780):1614-20.
9. Danecek P, Auton A, Abecasis G, Albers CA, Banks E, DePristo MA, et al. The variant call format and VCFtools. Bioinformatics. 2011;27(15):2156-8.
10. Risso DS, Mezzavilla M, Pagani L, Robino A, Morini G, Tofanelli S, et al. Global diversity in the TAS2R38 bitter taste receptor: revisiting a classic evolutionary PROPosal. Sci Rep. 2016;6:25506.
11. Heike Cea. Single Nucleotide Polymorphism Discovery in

TBX1 in Individuals with and without 22q11.2

Deletion Syndrome. Birth Defects Research (Part A). 2010;88(1):54-63.
12. Saitou N YF. Evolution of primate ABO blood group genes and their homologous genes. Mol Biol Evol. 1997;14(4):399-411.
13. Goeury T, Creary LE, Brunet L, Galan M, Pasquier M, Kervaire B, et al. Deciphering the fine nucleotide diversity of full HLA class I and class II genes in a well-documented population from sub-Saharan Africa. HLA. 2018;91(1):36-51.
14. Hedrick PW, Thomson G. Evidence for Balancing Selection at Hla. Genetics. 1983;104(3):449-56.
15. Bryk Jea. Positive selection in East Asians for an EDAR allele that enhances NF-kappaB activation. . PLos One. 2008;3(5).
16. Bersaglieri T, Sabeti PC, Patterson N, Vanderploeg T, Schaffner SF, Drake JA, et al. Genetic signatures of strong recent positive selection at the lactase gene. American Journal of Human Genetics. 2004;74(6):1111-20.
17. Reed FA, Akey JM, Aquadro CF. Fitting background-selection predictions to levels of nucleotide variation and divergence along the human autosomes. Genome Res. 2005;15(9):1211-21.
18. Korneliussen TS, Moltke I, Albrechtsen A, Nielsen R. Calculation of Tajima's D and other neutrality test statistics from low depth next-generation sequencing data. BMC Bioinformatics. 2013;14(289):289.
19. Hughes DA, Tang K, Strotmann R, Schoneberg T, Prenen J, Nilius B, et al. Parallel selection on TRPV6 in human populations. PLoS One. 2008;3(2):e1686.

20. Barrett JC, Fry B, Maller J, Daly MJ. Haploview: analysis and visualization of LD and haplotype maps. Bioinformatics. 2005;21(2):263-5.
21. Larkin MA, Blackshields G, Brown NP, Chenna R, McGettigan PA, McWilliam H, et al. Clustal W and Clustal X version 2.0. Bioinformatics. 2007;23(21):2947-8.
22. Bhatia G, N. Patterson, et al. . Estimating and interpreting F-ST: The impact of rare variants. Genome Res. 2013;23(9):1514-21.
23. Smith JM, Haigh J. The hitch-hiking effect of a favourable gene. Genet Res. 1974;23(1):23-35.
